# Acknowledgement to Reviewers of *Materials* in 2017

**DOI:** 10.3390/ma11010110

**Published:** 2018-01-11

**Authors:** Materials Editorial Office

**Affiliations:** MDPI AG, St. Alban-Anlage 66, 4052 Basel, Switzerland

Peer review is an essential part in the publication process, ensuring that *Materials* maintains high quality standards for its published papers. In 2017, a total of 1483 papers were published in the journal. Thanks to the cooperation of our reviewers, the median time to first decision was 18 days and the median time to publication was 44 days. The editors would like to express their sincere gratitude to the following reviewers for their time and dedication in 2017:
Aaleti, SriramLianos, PanagiotisAasmundtveit, Knut E.Liao, Chien-SenAbdelal, G.Liao, Ying-ChihAbdelaziz, Sherif LotfyLiaparinos, Panagiotis F.Abdelgaied, AbdellatifLicheri, RobertaAbdul Samad, YarjanLichołai, LechAbdul-Aziz, AliLichtenegger, HelgaAbduo, JaafarLiebscher, MarcoAbendroth, MartinLiefeith, KlausAbot, JandroLiew, Jat-Yuen RichardAbousnina, RajabLignola, Gian PieroAbramovich, HaimLikodimos, VlassiosAcet, MehmetLim, Young SooAchilias, Dimitris S.Lim, JoonwonAdachi, TakujiLima, CarmineAdam, WittekLi-Mayer, JoannaAdjaoud, OmarLin, Kuan JiuhAdumitroaie, AdiLin, ZhibinAeberhard, UrsLin, Chao-SungAfanasiev, P.Lin, Hsiun-KaiAfonin, KirillLin, Dan-JaeAfroughsabet, VahidLin, Tai-YuanAfshinnia, KavehLin, King-FuAgarwal, MangilalLin, HungyenAggelis, DimitriosLin, Chih-LangAgrafioti, AnastasiaLin, Gong-RuAgrawal, Kumar VaroonLin, Chi-ChangAgrela, FranciscoLin, Chih-LungAgrios, AlexanderLin, CPAguiar-Ricardo, AnaLin, Chun-MingAhmad, Hafiz WaqarLin, Jong-LiangAhmad, FayyazLin, Jau-WenAhmmed, K.M. TanvirLin, Han ChienAhn, Kyung HyunLin, I-NanAhn, JaehunLing, MauriceAhn, Kwang-HyunLinß, SebastianAikawa, ShinyaLionetto, FrancescaAkanji, LateefLiopo, AntonAkbar, SheikhLiparoti, SaraÅkermo, MalinLisiecki, AleksanderAkhavan, BehnamLisovenko, DmitryAkitsu, TakashiroLiss, Klaus-DieterAkkutlu, I. YucelLISSORGUES, GaëlleAkm, NewazLitina, ChrysoulaAksamija, ZlatanLitti, LucioAkyol, FatihLittle, Brenda J.Akyurtlu, AlkimLiu, AllenAlam, Md. MehboobLIU, K.Alam, ShahriaLiu, GangAlam, TalukderLiu, PeiAlam, Mohammad ParvezLiu, Sung-PoAlapan, YunusLiu, RenAlasbahi, BandarLiu, PengAlberti, AlessandraLiu, QingfanAlbizuri, JosebaLiu, YijinAlcoutlabi, MatazLiu, YunlingAlcubilla, RamónLiu, JinyunAlegre, CinthiaLiu, AiqinAlema, FikaduLiu, Fwu-HsingAlessandri, IvanoLiu, Xian HuAlhalawani, AdelLiverani, LilianaAli, MajidLizio, GiuseppeAlina, AlinaLlanes, LuisAljankawey, Abdualah S.Lloret, FernandoAljazaeri, Zena R.Lo, Chien-fongAlly, Moonis RazaLo Re, GiadaAlMangour, BandarLobato, JustoAlmeida, Maria Adelaide AraújoLodewijks, KristofAlmeida, Henrique De AmorimLogo, JanosAlmodovar, JorgeLolla, DineshAlonso, JavierLombardi, JohnAl-Sibahy, AdnanLong, JérômeAltomare, MarcoLongoni, GianlucaAltstadt, EberhardLópez, G.A.Altstädt, VolkerLópez De Lacalle, Luis NorbertoÁlvarez, José IgnacioLópez De Lacalle, L.NorbertoAlvear, DanielLopez-Crespo, PabloAlves, MartaLópez-Garzón, F.J.Aman, SergejLópez-Lorente, Ángela InmaculadaAmaro, A.M.Lops, DiegoAmbrosio, LuigiLorenz, MichaelAmeri, Shideh K.Lorenz, AlexanderAMIGÓ, VICENTELorenzo, MiguelAminYavari, S.Losic, DusanAmmu, PrhashannaŁosiewicz, B.An, JiaLove, RobertAnagnostopoulos, Costas A.Loveridge, MelanieAnastasova, SalzitsaLow, It MengAnderson, Wayne A.Lozano-Guerrero, Antonio JoséAnderson, Travis M.Lu, ChenyangAndreoli, EnricoLu, GeyuAndreou, ChrysafisLu, LiAndrukhov, OlehLuber, SandraAndrzejewska, EwaLucenti, ElenaAngelici, Robert J.Łuczak, SergiuszAngelini, EmmaLughofer, EdwinAnim-Danso, EmmanuelLuk, Ting S.Annamalai, Pratheep KumarLukashev, PavelAnnamdas, VenuLukasz, BorowikAnnamdas, Venu G.M.Łukaszewicz, MikolajAnnunziata, MarcoŁukomksa-Szymańska, MonikaAnselmi-Tamburini, UmbertoLumay, GeoffroyAntonio, APICELLALundstrom, MarkAntoniotti, SylvainLungu, Claudiu T.Antonov, StoichkoLuo, ShudongAntunes, ElsaLuong, Dzung DinhAnyalebechi, PrincewillLupan, OlegAnyszka, RafałLusi, MatteoAoyagi, KentaLuukkonen, TeroAphale, AshishLu-Yin, LinApollonio, CiroLuzi, FrancescaAppadoo, Srimantoorao S.Lyeo, Ho-KiArabnejad, HadiLyons, John G.Araña-Mesa, JavierŁyszkowski, RadosławAraromi, OluwaseunLyutakov, OleksiyArena, FrancescaMa, JinxingArevalo, CristinaMaalouf, ChadiArgia, KatsuhikoMacaluso, RobertoArias-Gonzalez, FelipeMackinnon, Ian D.R.Arnaud, FrançoiseMaçôas, ErmelindaArnold, Donna C.Maczka, MirosławArrua, DarioMadala, SrikanthArteiro, AlbertinoMadasamy, ThangamuthuArtero Guerrero, Jose AlfonsoMadge, ShantanuArtetxe, BeñatMaeda, HirotakaArvapally, Ravi KumarMaeder, XavierAryan, PouriaMaegawa, SatoruAsamoto, ShingoMaekawa, KoichiAshekuzzaman, S.M.Magnaldo, ThierryAshraf, WardaMagyari, KlaraAshruf, ColinMahajan, SahilAssender, HazelMahata, Manoj KumarAstarita, A.Mahjoub, RezaAta, SeisukeMahoutian, MehrdadAtabaev, TimurMaia, Lino M.Atabaev, Timur Sh.Maiberg, MatthiasAthanasiou, ChristosMaier, PetraAttar, HooyarMaio, AndreaAttia, Mondher HelmiMaiwa, HiroshiAttik, NinaMakarona, E.Atwood, Sara A.Mäkelä, Jyrki M.Auffermann, GudrunMakov, GuyAuger, Maria AMalfait, Wim J.Auppatham, NakarukMalgorzata, WisniewskaAvendano-Franco, GuillermoMallavia, R.Ayala, JuliaMallidi, SrivalleeshaAymard, LucMaloy, Stuart A.Aymerich, F.Mamidala, RamuluAZNÁREZ GONZÁLEZ, JUAN JOSÉMan, DulaAzoug, AurelieManakari, VyasarajBachaga, TarekMandapati, Ramakrishnam RajuBae, ChangdeuckMander, JohnBae, SungchulMang, TiaBae, YoungchulMangialardi, TeresaBaeza Labat, Juan AntonioManna, Rudra SekharBagby, MichaelMansoori, G. AliBagdziunas, GintautasMansour, RabihBahk, Je-HyeongManukyan, Khachatur V.Bai, YangManzani, DaniloBaino, FrancescoManzhos, SergeiBajda, TomaszManzone, MarcoBaji, AvinashMao, YuanbingBajorek, AnnaMao, ChuanbinBajwa, Dilpreet S.Mao, YiminBajwa, RizwanMarais, StephaneBaker, JamesMarcadon, VincentBaker, PaulMarcellini, MorenoBalakrishnan, G.Marcinkevicius, SauliusBalanay, Jo Anne G.Marczak , JacekBalandraud, XavierMardare, Andrei I.Balázsi, CsabaMarie, MohammedBalcerzak, MateuszMariniello, LoredanaBaldo, NicolaMariusz, KubusBalke, BenjaminMarken, FrankBallesta-Claver, JulioMarkopoulos, AngelosBalogh, AttilaMarmiroli, NelsonBaltakys, KestutisMarmy, PierreBaltazart, VincentMarques, Carlos Alberto FerreiraBaltriukiene, DaivaMarquis, Damien M.Ban, MasahitoMarradi, AlessandroBanach, MarcinMarrs, Michael A.Bandyopadhyay, SulalitMartellotta, FrancescoBanham, DustinMartí Albiñana, José V.Bani, DanieleMartin, Van De VenBányai, TamásMartínez Pañeda, EmilioBarbier, A.Martinez-Calvo, MiguelBarbillon, GrégoryMartínez-Máñez, RamonBarbosa, JoaquimMartino, LucaBarbot, Jean FrancoisMartino, SabataBardaweel, HamzehMartín-Ramos, PabloBarile, ClaudiaMartins, RodrigoBarker, Philip J.Martone, AlfonsoBarlage, Douglas W.Martowicz, AdamBarmatov, EvgenyMaruf, Sajjad H.Barra, GiuseppinaMarvasi, MassimilianoBarrau, SophieMarx, MichaelBarretta, RaffaeleMasato, UedaBarriobero-Vila, PereMascia, LenoBartocci, PietroMassera, JonathanBarucca, GianniMasuda, YoshitakeBaryshnikov, Sergey VasilevichMatanovic, IvanaBasaran, CemalMaterer, Nicholas F.Basaran, OsmanMateusz, KoziolBasiak, EwelinaMathaudhu, SuveenBassani, PaolaMathew, NithinBäßler, RalphMathew, MathewBastidas, David M.Mativenga, MalloryBattabyal, ManjushaMatko, VojkoBauchy, MathieuMatsubara, MasahikoBaudis, StefanMatsuda, TokiyoshiBaumann, Felix W.Matsuda, YasuhiroBaumeister, JoachimMATSUGAKI, AiraBaumgarten, MartinMatsumoto, AkikazuBayer, Ilker S.Matsuo, TetsujiBaykal, DorukMatsuoka, TsuneyoshiBayraktar, E.Matsuura, KazunoriBechelany, MikhaelMatsuyama, KiyoshiBechou, LaurentMatsuzaki, KorenobuBéchou, LaurentMatsuzaki, YoshioBecker, ThorstenMatzeu, GiusyBedon, ChiaraMauksch, MichaelBegines, BelenMaurel, LauraBehnood, AliMauriello, FrancescoBehrens, Bernd-ArnoMaurotto, AgostinoBehrou, RezaMavrogonatou, EleniBella, FedericoMazahery, A.Bellini, CostanzoMazari, AdnanBelver, CarolinaMazeika, LiudasBelviso, ClaudiaMazurek, GrzegorzBelyakov, AndreyMazzio, Katherine A.Ben Sedrine, NebihaMbey, Jean AimeBencardino, FrancescoMcAndrew, Anthony R.Bender, FlorianMcCloy, JohnBendersky, Leonid A.McDonald, ArmandoBenedetti, IvanoMéausoone, Pierre-JeanBenetti, Ana RaquelMedvedovski, EugeneBenseman, Timothy M.Meenashisundaram, Ganesh KumarBenvenuti, ElenaMehrali, MehdiBerardi, Valentino PaoloMehrpouya, MehrshadBeretta, StefanoMejia, IsraelBerge, FranzMekonnen, Tizazu H.Bergum, KristinMelchers, Robert E.Berlanga, CarlosMelchionna, MicheleBermudo, CarolinaMelro, António RuiBernardello, FabioMelvin, Gan Jet HongBernardo, LuísMemarzadeh, KavehBernards, MatthewMen, YongjunBernède, J.C.Menabue, LediBernini, RomeoMéndez, BianchiBerski, WiktorMendis, ChaminiBerthebaud, DavidMeneghetti, GiovanniBerthod, PatriceMenendez, RosaBertino, Massimo F.Menezes, PradeepBerto, FilippoMeng, XinBertoncello, PaoloMeng, LeBertrand, CharrierMeng, FanqiangBesara, TigletMeng, QingenBetancur, RafaelMeng, LingqiangBetelu, StéphanieMengucci, PaoloBeuerle, FlorianMenna, CostantinoBeves, JonathonMensah, Enoch A.Beyerlein, Irene J.Mergia, KonstantinaBhatt, AnandMerle, BenoitBhattacharjee, TilakMerodio, JoseBhattacharya, MrinalMesarovic, SinisaBiacchi, AdamMesbah, AdelBialecka-Florjańczyk, EwaMetz, RenaudBiałobrzeska, BeataMey, JörgBidola, Pidassa M.Miani, FabioBiernat, JanMiao, JiashiBiesuz, MattiaMiao, GuoxingBihamta, RezaMichalcová, AlenaBiles, William ErnestMichalska-Domańska, MartaBilic, AnteMichaud-Soret, IsabelleBiranchi, PandaMicheli, DavideBirkett, MartinMickelson, Alan R.Birloaga, IonelaMickelson, AlanBishay, PeterMickovski, Slobodan B.Bisio, FrancescoMigita, SatoshiBisker, GiliMiguel, Castro-ColinBisoyi, Hari KrishnaMihara, TsuyoshiBiswas, ShaurjoMikhailova, AlexandraBlanco, IgnazioMiklaszewski, AndrzejBlanco-Valera, Maria TeresaMilanese, ChiaraBlanloeuil, PhilippeMilanesio, MarcoBlayo, AnneMilczarek, GrzegorzBlel, WalidMilia, EgleBloh, Jonathan Z.Milkereit, BenjaminBlomme, ErikMilovanovic, BojanBochentyn, BeataMin, Kyung-SanBochud, NicolasMinakshi, ManickamBoffa, VittorioMinami, IchiroBogan, JustinMinea, Alina AdrianaBogdanchikova, NinaMines, R.A.W.Boland, ConorMines, Robert A.W.Bolander, JohnMingareev, IlyaBoldrini, StefanoMingler, BernhardBolotov, LeonidMingo, BeatrizBolvin, HélèneMiniaci, MarcoBolz, SebastianMiniewicz, AndrzejBombac, AndrejMiranda Guedes, RuiBon, VolodymyrMiranda Salvado, Isabel MargaridaBonek, MirosławMiras, Haralampos N.Bong, Hyuk JongMirdamadi, AlirezaBonifacio, AloisMiri, Amir K.Boone, Matthieu N.Miron, Richard J.Borbas, K. EszterMiroshnichenko, AndreyBorchers, C.Mirsayar, MirMiladBoria, SimonettaMirzadeh, H.Borkar, TusharMisaelides, PanagiotisBorkow, GadiMishra, YogendraBorkowski, LeszekMishra, RajeshBormashenko, EdwardMitsuhiro, TerakawaBorsacchi, SilviaMiura, SeijiBosch, Rik-WouterMiyamoto, ManabuBose Goswami, SanjuktaMiyauchi, MasahiroBosia, FedericoMochalin, VadymBossuyt, FrederickModigell, MichaelBouazizi, NabilMoeini Sedeh, MahmoudBoulanger, Joan A.R.Mohamed, WalidBoumerzoug, ZakariaMohammad, ArjmandBouropoulos, NikolaosMohammed, Ghusoon RidhaBoyer, MichaelMohanchandra, KotekarBradley, JamesMOHANTA, ANTARYAMIBrando, GiuseppeMohtadi Bonab, Mohammad AliBrandt, MilanMoineau-Chane-Ching, Kathleen I.Branquinho, Rita Maria Mourão SalazarMokaya, RobertBrantley, William A.Mokhtari, OmidBrault, J.Molenda, JaninaBraymand, SandrineMølhave, KristianBredol, MichaelMolina, Carmen BelénBrenna, StefanoMolina-Aldareguía, Jon MikelBresser, DominicMollón, VictoriaBrigham, KatharineMonchau, FrancineBrighenti, RobertoMonetta, TullioBrink, TobiasMonje, AlbertoBrischke, ChristianMONOT-LAFFEZ, IsabelleBrnic, JosipMontanari, R.Brögelmann, T.Montealegre-Melendez, IsabelBrokmeier, Heinz-GünterMonteil-Rivera, FannyBrostow, WitoldMontero-Chacón, FranciscoBrudzynski, KatrinaMontiel, VicenteBrunner, AndreasMontufar Jimenez, Edgar BenjaminBrychcy-Rajska, EwaMontuori, RosarioBrzeziński, MarekMonzón, Mario D.Bu, JunfuMoon, Doo KyungBuccolieri, AlessandroMoon, Hong ChulBucha, JozefMoon, JihoBuckley, David. J.Moon, JuhyukBuckman, JimMoore, Axel C.Bucur, VoichitaMoore, MoretonBueno, MoisesMoraczewski, KrzysztofBufo, Sabino A.Moramarco, VincenzoBuha, JokaMorasch, AlexanderBuhan, Patrick DeMorcillo, M.Buhl, Josef-ChristianMore, Karren LeslieBui, Tinh QuocMoreno, G. GarciaBulgariu, LauraMoreno Arboleda, Francisco JavierBulic, MladenMoreno-Atanasio, RobertoBull, SteveMoreno-Maroto, José ManuelBulou, HervéMoretti, LauraBundesmann, C.Morgado, JorgeBünzli, Jean-ClaudeMoriconi, GiacomoBuonocore, FrancescoMorikawa, AtsushiBurbank, MalcolmMorley, Nicola A.Burgert, IngoMoroz, AdamBurghard, ŽaklinaMorreale, MarcoBurin, Alexander L.Morrison, Finlay D.Burkel, EberhardMosallaei, HosseinBurnat, BarbaraMosey, StephenBurrows, NathanMoskal, G.Buscarnera, GiuseppeMosnackova, KatarinaBusche, ChristophMostafa, AhmadBüsselberg, DietrichMoura, Maria J.Butt, JavaidMourdikoudis, StefanosButylina, SvetlanaMoure, AlbertoBuyle-Bodin, FrancoisMoussaoui, KamelBychanok, DzmitryMouzakis, Dionysios E.Bylski-Austrow, Donita I.Mozurkewich, GeorgeCabal, BelénMróz, WaldemarCaballero, AlvaroMrukiewicz, MateuszCabanelas, Juan CarlosMueller, Peter P.Cabeza, M.Muench, FalkCabrales, LuisMuenzenberg, MarkusCacciato, GiuseppeMuhunthan, BalasingamCachim, PauloMukherjee, PinakiCacialli, FrancoMuley, PranjaliCaggiano, AntonioMüller, AndreiCai, FengMünch, AlexanderCai, ZhongyuMunir, KhurramCai, LipingMuniz-Miranda, FrancescoCaiazzo, FabriziaMuñoz, CarlosCalahorra, YonatanMunz, Richard J.Calero, Olga CaballeroMünzenrieder, Niko StephanCall, MindyMurata, JunjiCallahan, Laura SmithMurdoch, Billy JamesCalvo-Guirado, José LuisMurugan, N. ArulCambronero, Luis Enrique G.Muscio, AlbertoCamerlingo, CarloMuthukumar, SriramCametti, C.Mutyala, KalyanCamillo, D’ArcangeloMyers, OliverCammino, Roberto A.Mylvaganam, KausalaCamões, AiresMynbaeva, MarinaCampbell, JohnNa, KunCamposeo, AndreaNabae, YutaCampuzano Ruiz, SusanaNadolna, JoannaCancelliere, PiergiacomoNag, ManojCandolfi, ChristopheNagai, HirokiCannone Falchetto, AugustoNagaoka, MasatoCao, WeiNagaoka, ToruCao, LijunNagareddy, V KarthikCao, ZhenNagase, TakeshiCapdevila, CarlosNAGASHIMA, KazukiCapiglia, ClaudioNagatani, NaokiCapineri, LorenzoNagoshi, TakashiCaporali, StefanoNagy, EndreCarabineiro, Sonia A.C.Nair, Jijeesh R.Carabineiro, Sónia A.C.Nair, RemyaCarboni, MicheleNair, DevathaCardoso, DavidNakada, NobuoCarette, JérômeNakaharai, ShuCarlen, Edwin T.Nakahata, KazuyukiCarli, StefanoNakano, TamakiCarlone, PierpaoloNakata, T.Carosio, FedericoNakielski, PawelCarrado, AdeleNam, Ki-WooCarro, DiegoNan, YanghaiCartagena-Rivera, AlexanderNanami, NorimichiCarter, AlanNanda, HimansuCasado-Coterillo, ClaraNandhakumar, IrisCasalino, GiuseppeNandi, DebaleenaCaschera, DanielaNandwana, VikasCasey, SeanNanjo, KazuyoshiCasey, AlanNaoe, TakashiCassidy, JohnNapolano, LoredanaCastagne, SylvieNARA, HirokiCastle, ElinorNara, YoshitakaCastro, AndreNarayanan, ShankarCasula, MicheleNarayanan, GaneshCatargi, BogdanNaresh-Kumar, GunasekarCattoën, XavierNaser, M.Z.Cavallotti, PietroNasrollahi, AmirCawley, PeterNatsui, ShungoCazzanelli, MassimoNatsuki, ToshiakiCeccone, GiacomoNattestad, AndrewCech, VladimirNavarro, CarlosCelada-Casero, CarolaNavarro, JoseCelano, UmbertoNavarro, Francisco JavierCenteno, Teresa A.Navarro, María VictoriaCeretti, MonicaNavas, JavierCerny, MiroslavNaviglio, SilvioCerro-Prada, ElenaNayak, ArunimaCesano, FedericoNazarov, Andrej P.Chady, TomaszNazin, GeorgeChaim, RahmanNazir, MianChaisakul, PapichayaNěmeček, JiříChakrapani, Sunil KishoreNemes, Norbert M.Chalioris, ConstantinNemestothy, NandorChan, CHI WAINeri, PaoloChan, Kwai S.Netravali, AnilChandrappan, JayakrishnanNetskina, OlgaChang, HoNeuenschwander, JuergChang, Chi-jungNeumeister, PeterChang, Chih-YuNewman, J.C., Jr.Chang, Chang-TangNg, Ching-TaiChang, Wei-JenNganga, SaraChang, Yao-FengNgo, Chi-VinhChang, Fuh-YuNguyen, TuyenChang, Ting-ChangNicholson, John W.Chantana, JakapanNico, LanghofChao, JesusNicolau, AnaCharilaou, MichalisNicoletta, Fiore PasqualeChass, Gregory AdamNie, MengyanChau, Lai-KwanNiedermann, PéterChaubey, AnilNieminen, KaarloChavez, FermanNien, Yung-TangChe, Fa XingNieslony, PiotrChembo, Yanne K.Nikas, George K.Chemenda, AlexandreNikishin, SergeyChen, Yuan-TsungNikkhah-Moshaie, RoozbehChen, Pang-ShiuNikolelis, Dimitros P.Chen, TianyiNimbalkar, SanjayChen, MingzhouNishihara, MasamichiChen, YunpengNishiyama, NorikazuChen, I. WenNisticò, RobertoChen, BiaoNittami, TadashiChen, ChunhuiNiu, Li-BinChen, MingshengNiwano, MichioChen, QuarkNix, WilliamChen, Po-YuNobile, LucioChen, Ruei-SanNobuyuki, IWATAChen, Lung-ChienNogaret, Alain R.Chen, JunNogueira Perez, Juan JoseChen, Teng-MingNommeots-Nomm, AmyChen, KaiNomura, TakahiroChen, Jer-MingNonappa, NonappaChen, ShuoNorouzzadeh, PayamChen, Hui-YuNorvez, SophieChen, Tao-HsingNothdurft, FrankChen, ChuantongNotta-Cuvier, DelphineChen, HailongNottrodt, NadineChen, Bor-kuanNouh, MostafaChen, Jung-HsuanNouri, NimaChen, Hsien-YehNovak, PetrChen, FanNovák, IgorChen, JiaNowak, AndrzejChen, Zhi-GangNowak, SaschaChen, Chiing-ChangNtalli, Nikoletta G.Cheng, XingguoNulwala, HunaidCheng, Yuh-JenNúñez-Delgado, AvelinoCheng, Chih-ChiaNurrohman, HamidCheng, Ko-TingNurul Fazita, M.R.Cheng, Yang-TseNussbaumer, AlainCheng, GuangO’Donoghue, Padraic E.Cheng, Chun-HuObraztsov, AlexanderCheng, TaoOcchipinti, LuigiCheng, AnOchalski, TomaszCherevan, AlexeyOchiai, TsuyoshiCherman, VladimirOćwieja, MagdalenaCherukara, MathewOehlschlaeger, MattChesneau, XavierOehring, M.Chiang, Anthony S.T.O'Flaherty, FinChiang, Long Y.Ogino, HirakuChiara, MandolfinoOgorek, RafalChicardi, ErnestoOh, Min-WookChidambareswarapattar, ChakkaravarthyOh, TeresaChien, Feng-TsoOh, Sang SoonChieruzzi, ManilaOh, Soong JuChih, Chieh HsuOhno, SeigoChilkoor, GovindaOjard, GregChitale, KedarOjovan, MichaelChitrakar, SaileshOk, Myoung-RyulChiu, WingOk, Jong G.Chiu, ChunOkada, GoChiu, Tien-LungOkada, NarihitoChladek, GrzegorzOkamoto, MasamiChmielarz, LucjanOkawa, SeigoChmielewska, EwaOkhay, Olena I.Chmielus, MarkusOkitsu, YoshitakaCho, Dong-WooOlchowka, JacobCho, Jae UngOliveira, João PedroCho, Sung M.Oliveira, GustavoCho, ByungjinOlivera-Pastor, PascualChoi, SukwonOlivier, MonginChoi, WonjoonOlmos, M. ElenaChoi, HeungjaeOlofinjana, AyodeleChong, William W.F.Olson, Sandra L.Chong, AlvinOluwafunmilayo, JosephChopineau, JoëlOng, WernChou, Hsien-TerOono, NaokoChoudhuri, DeepOpielak, MarekChow, LouisOpitz, JörgChoy, Wallace C.H.Orbulov, ImreChoy, Young BinOrcajo, GiselaChristofidou, Katerina A.Ordoñez, CelestinoChroneos, AlexanderOrejon, DanielChruściel, Jerzy J.Ortega, NaiaraChu, ShidongOrtega, J. MarcosChu, JiaxiangOswald-Tranta, BeateChung, Yeong-JinOta, HirokiChung, Kyung YoonOterkus, ErkanChung, Yong-AnOttonelli, MassimoChung, Chul-WooOttria, LilianaChung, Sheng-HengOujja, MohammedChung, Kwok-hungOzaki, ShingoChurch, Benjamin C.Ozaki, RyotaroCicala, GianlucaOzbakkaloglu, TogayCicciu, MarcoÖzyürek, MustafaCifelli, MarioPablos, CristinaCifuentes, Sandra C.Pachego-Torgal, FernandoCiliberto, SergioPadalkar, MugdhaCiofani, GianniPadhy, GirishCioffi, NicolaPaegle, IevaČiplys, DaumantasPaesani, FrancescoCizek, JanPagnotta, LeonardoClaramunt, JosepPahlevani, FarshidClarke, RonPainter, RogerClarke, KesterPaja̧ķ, PaulinaClaßen, MartinPalasantzas, GeorgeClay, R.T.Palchoudhury, SoubantikaClayton, John D.Palka, NorbertClyburne, JasonPalumbo, GianfrancoCoates, Nelson E.Pan, ZengxiCodispoti, RosamariaPan, YayueCoehoorn, R.Pan, Guan-TingCoffey, KevinPanagiotis, SpathisColangelo, FrancescoPandarese, GiuseppeColegrove, PaulPandolfi, AssuntaColeman, NicholaPang, Eun-KyoungCollings, Ines E.Panomsuwan, GasiditCollins, Maurice N.Panoutsos, GeorgeCollins, Peter C.Pantazopoulos, George A.Colomines, GaëlPantazopoulos, GeorgeContreras-Cáceres, RafaelPanteghini, AndreaCorcuera, Maria AngelesPanwisawas, ChinnapatCórdoba, José ManuelPaolone, AnnalisaCorezzi, SilviaPap, ZsoltCormack, Alastair N.Papaccio, GianpaoloCornell, B.Papadakis, RaffaelloCoronas, JoaquínPapadimitriou, Sofia A.Corpas Iglesias, Francisco AntonioPapaefthymiou, SpyrosCorradi, MarcoPapageorgiou, George Z.Correia, José A.F.O.Papagiannopoulos, AristeidisCorreia, António Alberto SantosPapaioannou, Evangelos ThCortes Corberán, VicentePapia, EvaggeliaCosma, PinalysaPapoulis, DimitriosCosta, RubénPapula, SuviCostanza, GirolamoParadiso, ValentinoCoudert, François-XavierParajuli, DurgaCox, SophieParaskevas, DimosCozzoli, P. DavidePardanaud, CédricCozzolino, AnthonyParigger, Christian G.Cramer, TobiasParisi, SalvatoreCrane, DouglasParisi, CristianCrassous, Jérôme J.Park, Jang MinCrawford, SeanPark, WansuCrawford, Thomas M.Park, SangmoonCrean, AbinaPark, MinjoonCremonesi, MassimilianoPark, JaehoonCress, Cory D.Park, Jea-gunCrisan, BogdanPark, Sun HongCristiani, PierangelaPark, Chan HumCrivelli, DavidePark, Jun-SangCroatt, Mitchell P.Park, Jin-SeongCroes, KristofPark, Hyung-hoCrupi, VincenzoPark, Yong IlCsík, AttilaPark, Jong-SeokCucciniello, RaffaelePark, Myoung-HwanCuesta, I.IvánPark, Kyeong-WonCui, HaitaoPark, Joo-CheolCui, ZhibinPark, Nam-GyuCuitiño, Alberto M.Park, K.Cutroneo, MariapompeaPark, Jang-UngCzapik, PrzemysławParkes, MariaCzech, BożenaParrilli, AnnapaolaCzujko, TomaszParsons, J.G.D’Agostino, StefaniaParteli, EricDa Costa, Ana RosaPasculli, AntonioDa Costa, PatrickPascuta, PetruDabiri, DanaPasetto, MarcoD'accolti, LuciaPassariello, ClaudioDahal, TulashiPasseri, DanieleDalessandri, DomenicoPasserone, AlbertoD'Alessandro, AntonellaPasternak, ElenaDammaschke, TillPasti, LuisaDa'na, EnshirahPastor, José-YgnacioDang, CuongPastore, CarloDang, AleiPastore, RobertoDanno, AtsushiPaszkiewicz, SandraDappe, Yannick J.Patel, SaurinDarmanin, ThierryPatel, VikasDart, AndrewPatel, Rajen B.Darvell, BrianPato Doldan, BreoganDarvell, Brian W.Patra, AnirbanDas, OisikPauer, WernerDas, SanjibPaul, S.Das, JagotamoyPaul, Titan C.Dasgupta, SrimoyeePaul, ShubhajitDasgupta, GautamPawlus, PawelDatcheva, MariaPecht, Michael GerardDatta, JanuszPedraza, Óscar De La IglesiaDatta, RupsaPedzich, ZbigniewDauscher, AnnePeet, M.J.Daver, FugenPei, ZingwayDavies, IanPeiris, T.A. NirmalDavis, ClairePelled, GadiDavoudinejad, AliPellegrini, NicolaDawes, JudithPellegrino, FrancescoDawson, RichardPellejero, IsmaelDe Aza, PiedadPelletier, MathewDe Filippis, LuigiPellicer, TeresaDe Finis, RosaPeng, Shuhuade Hoop, CornelisPenney, David J.De La Prida, Victor ManuelPereira, LuizDe Los Santos, Desiree M.Pereira De Oliveira, Luiz AntonioDe Pertis, MarcoPerepelyuk, MarynaDe Sio, LucianoPERETTI, RomainDe Sousa Meneses, DomingosPérez, GloriaDe Wild, JessicaPérez, SoledadDecaluwe, Steven C.Pérez, P.Decker, SabinePerez-Tomas, AmadorDecroly, AndréPérigo, Élio AlbertoDe-Juan-Pardo, Elena M.Peron, MircoDel Hoyo, C.Perpiña, XavierDelaunois, FabiennePerrella, MicheleDelbe, KarlPerron, AurelienDelepine-Lesoille, SylviePerry, NicolaDelfanazari, KavehPersano, LuanaDelrue, StevenPersaud, S.Y.Delsaute, BricePersson, DanDemadis, Konstantinos D.Perteghella, SaraDemir, Ali GökhanPervaiz, SalmanDeng, HongbingPervak, VolodymyrDeng, HuaPeshek, TimothyDeniz, VedatPestryakov, AlexeyDepczynski, WojciechPeter, NgeneDeravi, Leila F.Peterka, PavelDeren, Przemyslaw J.Petrila, IulianDeringer, VolkerPettignano, AlbertoDermentzis, Konstantinos I.Pfeif, ErikDerrick, MottPham, Anh HoangDesmarets, ChristophePhan, HungDeville, SylvainPhillippa, BronsonDhakal, AshimPhung, Quoc TriDhakshinamoorthy, AmarajothiPiana, GiuliaDi Bartolomeo, AntonioPiana, GianfrancoDi Bella, GuidoPicard, DonaldDi Luccia, AldoPicollo, MarcelloDi Maggio, RosaPiconi, CorradoDi Pasqua, AnthonyPierron, Olivier N.Di Schino, AndreaPietri, SylviaDialami, NargesPietro, GaliziaDIAS DA COSTA, DANIELPietrzyk, BozenaDíaz De Tuesta, Jose LuisPiluso, SusannaDíaz Fernández, BelénPIN, Jean-MathieuDichiara, Anthony B.Pinchuk, AnatoliyDickens, TarikPineda, EloiDierre, BenjaminPineda, PalomaDimakis, NicholasPinto, DeesyDing, FeiPiotr, SzczepanskiDing, XinPisarska, JoannaDinischiotu, AncaPistidda, ClaudioDinjaski, NinaPistone, AlessandroDiroll, BenjaminPistorius, Petrus ChristiaanDirrenberger, J.Placido, FrankDitaranto, NicolettaPlakas, KonstantinosDixon, David A.Planas, JaimeDoi, KazuyaPlatt, PhilipDolgos, Michelle R.Podolsky, JosephDomaneschi, MarcoPoehl, FabianDomenici, ValentinaPoelman, DirkDomingo, ConcepciónPoerschke, UteDominguez, StephanePoggio, ClaudioDonato, LauraPogrebnjak, AlexanderDonchev, AlexanderPohl, MichaelDong, YalinPoinern, Gérrard Eddy J.Dong, Cheng-DiPojman, John A.Donnarumma, GiovannaPolanski, MarekDorman, JamesPolatidis, EfthymiosDoroudiani, SaeedPoletto, MatheusDorozhkin, SergeyPolimeni, AntonioDorri Moghadam, AfsanehPolimeni, AntonellaDotan, AnaPomi, RaffaellaDouali, RedouanePoon, JosephDow, Wei-PingPopescu, Andrei C.Drewniak, SabinaPopovich, V.A.Drexler, PetrPopuri, Srinivas R.Dreżewski, RafałPorada, SlawomirDrobek, MartinPorfiri, MaurizioDu, XushengPorojan, LilianaDu, HongjianPorras-Vazquez, Jose ManuelDu, YiPorter, DavidDuarte, IsabelPosada, ChrystianDuarte, Joao C.Poulikakos, LilyDubey, AshishPourrahimi, Amir MasoudDubey, ShriPourret, OlivierDucobu, FrançoisPowell, MichaelDuczek, SaschaPradeep, Konda GokuldossDudea, DianaPradhan, Sangram K.Dudina, DinaPrakash, AdithyaDuma, Virgil-FlorinPrasad, KarthikaDumas, JeraldPrasad, AnkushDumitrica, TraianPredoi, DanielaDumont, Pierre J.J.Predoi, Mihai V.Duncan, GreggPrevitali, BarbaraDunietz, Barry D.Price, Richard B.Duong, Hai M.Printz, Adam D.Durejko, TomaszProcek, MarcinDutil, YvanProchazka, IvanDutta, AniruddhaProt, Victorien EmileDutta, PoulamiProvine, J.Duty, ChadPrzestacki, DamianDuzhko, Volodimyr V.Przybylski, MarekDybała, JacekPu, HaihuiDyskin, ArcadyPudasaini, Pushpa RajDziedzic, AndrzejPuga, H.Dzimitrowicz, AnnaPuglia, DeboraDziubla, ThomasPuglisi, Francesco MariaEbara, RyuichiroPuiggalí, J.Ebong, AbasifrekePulati, NuerxidaEbrahimi, AmirPuli, VSEbrahimi, HamidPullano, SalvatoreEbramzadeh, EdwardPulugurtha, Markondeya RajEcheverría, AlbertoPuppo, FrancescaEchezarreta-López, Magdalena MagdalenaPurcell-Milton, FinnEdalati, KavehPurwar, RoliEden, Timothy J.Pustan, MariusEdmiston, PaulPyatina, TatianaEftekhari, MohammadrezaPyl, LincyEfthimiadou, Eleni K.Pyo, SukhoonEfthymiadis, P.Qaroush, AbdussalamEgorkin, Vladimir S.Qiu, JianhuiEhtemam Haghighi, ShimaQuagliotto, PierluigiEhtemam-Haghighi, ShimaQuintero, FélixEiser, ErikaQuock, RyanElhag, SamiRaabe, DierkEliyan, Faysal FayezRabari, RonilEllinas, KosmasRabizadeh, TaherEllmer, KlausRachmawati, DessyElm, Matthias T.Radecki, JerzyEl-Safty, Sherif A.Radek, NorbertElSawy, Ahmed H.Radenberg, MartinElshkaki, AymanRadi, EnricoEmadi, ArezooRǎducanu, DoinaEmmelmann, ClausRaedel, MichaelEndalkachew, Sahle-DemessieRaftopoulos, KonstantinosEndruweit, AndreasRaftopoulos, Konstantinos N.Engelberg, DirkRagab, KhaledEngler, OlafRahier, HubertEpifani, MauroRahman, ZiaurErb, UweRahman, ShafiqurErba, AlessandroRahman, MasudurErchiqui, FouadRahman, FaizErdem, EmreRahmani, RaminErick, OgamRaisutis, RenaldasErk, ErkRakhmonov, JovidErrico, Maria EmanuelaRamani, AlwarErwan, PaineauRamin, AghababaeiEsin, Vladimir A.Ramire-Castellanos, JulioEspinós, Juan PedroRamirez-Vick, JaimeEsposito, LucaRamsay, BruceEsqué-de Los Ojos, DanielRamulu, MamidalaEsquinazi, PabloRana, DipakEstefania Rojas-Hernandez, RocioRana, VijayEstellé, PatriceRane, G. KEstrade, SoniaRanganathan, NaliniEtacheri, VinodkumarRanzato, EliaEtienne, DagueRao, SmithaEyma, FlorentRao, AshitEymery, JoëlRao, HarishFaber, K.T.Rao, AshwinFabio, MazzaRaposo, MariaFacchini, FrancescoRapson, Trevor D.Falconi, ChristianRashid, R.Falentin-Daudré, CélineRaspanti, MarioFaleschini, FloraRatova, MarinaFalmbigl, MatthiasRau, Julietta V.Fan, ZhengRaucci, Maria GraziaFancey, KevinRazavi, JavadFang, FeiRe, RebeccaFang, ChangmingReddy, M.V.Fang, XiaolanReece, Michael J.Fantozzi, GilbertRegev, OrenFantuzzi, NicholasRegmi, BishnuFaraci, Francesca DaliaReilly, GwendolenFaraldos, MarisolReis, P.N.B.Farè, SilviaReis, LuisFarhat, SusanRen, QiangFaria, J.Ren, ZhenFarnood, Ramin R.Ren, HaoFaruqi, Mohammed A.Ren, BaiyangFaruqi, Mohamed A.Renner, FrankFasano, AndreaRenouf, MathieuFatyeyeva, KaterynaReshetilov, Anatoly N.Fauzi, IlianaRetegi, AloñaFavache, AudreyReverberi, Andrea P.Fawzy, Amr SherifRevuru, Rukmini SrikantFayaz, MohammadrezaReyes-Gasga, JoséFaye, OmarRhyee, Jong-sooFazal, AdnanRibeiro, Carlos SilvaFazeli, FatehRibeiro, José BarandaFedorko, GabrielRibeiro, Luís FrölénFedorov, PavelRibeiro, BettencourtFeld, ArturRicci, JohnFeldmann, MaikRicci, Pier CarloFelner, IsraelRicciotti, LauraFeng, Sheng-WeiRicco, RaffaeleFenn, MichaelRice, Cynthia AnnFernandes, IsabelRichard, CyrilleFernandes, Maria HelenaRicheton, ThiebaudFernandes, Maria Helena Figueira VazRicketts, NigelFernandes, Diana Mónica De Mesquita SousaRider, A.N.Fernandez, IgnasiRiedl, HelmutFernández, MartaRieker, ClaudeFernández Romero, Juan ManuelRiesen, HansFernandez-Gamiz, UnaiRigamonti, LucaFerns, GordonRimoldi, LucaFerrara, LiberatoRimondini, LiaFerrari, MaurizioRinaldi, AndreaFerreira, J.M.F.Rizal, ConradFerreira, Manuel MarquesRizza, GiancarloFerro, PaoloRizzo, PiervincenzoFerry, LaurentRoa, J.J.Fettkenhauer, ChristianRobert, DidierFezi, KyleRoberto, DominiqueFiedler, ThomasRochford, LukeFiglus, TomaszRockett, AngusFigueira, RitaRodriguez Navarro, JorgeFigueira, Rita BRodríguez-Alloza, Ana MaríaFigueiras, Fábio GabrielRodríguez-Bernaldo De Quirós, A.Figueiredo, MargaridaRoger, LeblancFilippo, RobertoRoggendorf, Matthias J.Finer, YoavRoggendorf, HansFink, KarinRogl, PeterFinkenstadt, VictoriaRohnke, MarcusFinkler, AmitRohwerder, MichaelFinšgar, MatjažRojas, SergioFintová, StanislavaRojas, Jose I.Fiore, VincenzoRokosz, KrzysztofFiorelli, JulianoRomán-Martínez, M.C.Fioretti, RobertoRomeira, BrunoFirlej, LucynaRomero, AldoFischer, HolgerRomero, PedroFischetti, Massimo V.Romero-García, VicenteFisher, BertinaRomero-Gómez, PabloFlandin, L.Romoli, LucaFleischmann, JonathanRopital, FrançoisFletcher, AshleighRoppolo, IgnazioFlood, NathanRosas, OmarFlorczyk, Stephen J.Rose, Joseph L.Floyd, RoyceRose, MarcusFocacci, FrancescoRosengren, BjörnFollain, NadègeRosenzweig, DerekFong, EileenRossella, FrancescoFonseca, JoanaRossetto, IsabellaForaboschi, PaoloRosspeintner, ArnulfForay, GenevieveRoszak, JoannaForcada, LaiaRotaru, AndreiForcellese, ArchimedeRotella, GiovannaForgan, RossRoters, FranzForget, AurelienRoth, JohannesFormela, KrzysztofRousakis, TheodorosForner, LeopoldoRousseau, BenoitFortenberry, RyanRoy, AnupamFoti, DoraRoy, SagarFrancesco Paolo, La MantiaRoy, SurajitFrancesconi, LucaRóżalska, BarbaraFrancisco, Montero-ChaconRtimi, SamiFranke, JörgRubio-Marcos, FernandoFranklin, EvanRudraraju, ShivaFrantziskonis, George N.Rudykh, StephanFraternali, FernandoRuff, AdrianFratini, FilippoRughoobur, GirishFreddi, FrancescoRühmer, DennisFrediani, PieroRuiz Raga, SoniaFreeman, Colin LRuiz-Rosas, Ramiro RafaelFrémont, HélèneRujikiatkamjorn, CholachatFrese, RaoulRunge, Jude MaryFressengeas, ClaudeRupérez, ElisaFreudenberger, JensRussell, Alan M.Freysoldt, ChristophRusso, SalvatoreFrigione, MariaenricaRusso, SaverioFrömling, TillRuys, Andrew J.Frontera, AntonioRůžek, RomanFrontera, PatriziaRyan, James G.Frostevarg, JanRyan, S.Fu, HankueiRyou, J.H.Fu, ZhezhenRyu, Sung-SooFu, ChenRyu, Sung HunFu, TengfeiRyu, Han-YoulFu, YongzhuRyu, Ja-HyoungFu, FengSaad-Eldeen, SaadFu, KunkunSaafi, MohamedFuh, JerrySabirov, IlchatFujii, SatoshiSaboori, AbdollahFujii, TakenoriSacco, RiccardoFujii, YoshiakiSacco, AdrianoFukuda, TakeshiSadao, AdachiFukumoto, MasahiroSadati, MonirosadatFukushima, JunSadeghifar, HamidrezaFukushima, TomohiroSadowski, LukaszFulvio, Pasquale F.Safavi, KamranFunato, MitsuruSafiuddin, Md.Furuhashi, AkihiroSafranski, DavidGabay, AleksandrSagebiel, John C.Gabdullin, NikitaSaha, ShyamaliGakhar, RuchiSaha, BivasGalán-Marín, CarmenSaha, BiswajitGalatus, RamonaSaharan, LalitaGalca, A.C.Sahariah, PriyankaGalindo-Rosales, Francisco J.Sahay, PeeyushGallagher, JohnSai, HiroakiGallego, AntolinoSaielli, GiacomoGallego, JuanSaikia, NabajyotiGaltieri, GiovannaSainidou, RebeccaGambarova, Pietro G.Sainoki, AtsushiGamsjäger, ErnstSaito, TakeshiGan, Chee KwanSaito, KazuyaGan, YixiangSaive, RebeccaGandji, Navid P.Sakai, JoeGao, JieSakai, MasatoshiGao, YunxiangSakairi, MasatoshiGao, WeiSakakura, MasaakiGao, WeiminSAKATA, Sei-ichiroGao, YanzheSalagnac, PatrickGaragnani, Gian LucaSalahub, DennisGarboczi, Edward J.Salaoru, IuliaGarbovskiy, YuriySaldana, ChristopherGarcia, RosarioSalerno, MarcoGarcia, AndresSalimi Jazi, MehdiGarcia, Jose E.Salinas Rodríguez, BeatrizGarcía, JuanSalmi, MikaGarcía, NuriaSalvador, Maria DoloresGarcía Gonzalo, Mª EsperanzaSalvo, AndreaGarcia-Escorial, AsuncionSamper, M.D.García-Márquez, AlfonsoSamperi, FilippoGarcía-Martínez, Jesús-MaríaSamuel, FawzyGarcía-Raffi, L.M.Sanati, MahdiGarcia-Segura, SergiSánchez, GoarGarcia-Tecedor, MiguelSánchez, MercedesGarcin, ThomasSánchez, Juan FranciscoGardelis, SpirosSánchez-Coronilla, AntonioGarg, NishantSánchez-García, DavidGaritaonandia, JoseSanchez-Herencia, Antonio JavierGarnier, GilSánchez-Soto, Luis L.Garrido, MarioSangiorgi, CesareGaska, KarolinaSanina, ViktoriyaGavioli, LucaSanli, AbdulkadirGe, AimingSanna, SimoneGe, LiyaSanson, AndreaGeiregat, PieterSansone, ClementinaGelbstein, YanivSantamarta, RubenGeletii, Yurii V.Santangelo, Paolo E.Genchi, GiadaSantecchia, EleonoraGeng, DonglingSantodonato, LouGentile, PiergiorgioSantoni, Marie-PierreGenty, EricSantos, AbelGeorge, Thomas F.Santos, Maria JacintaGeorgiou, E.P.Santos-Ruiz, LeonorGerard, MathiasSantschi, ChristianGervasio, Dominic F.Santulli, CarloGhabchi, RouzbehSanz-Herrera, Jose A.Ghafoori, ElyasSaoud, KhaledGhahremaninezhad, AliSapora, AlbertoGharaibeh, BurhanSarkar, DebanjanGhassemali, EhsanSarlin, EssiGheribi, Aimen.E.Saroglou, HarisGhidelli, MatteoSarvghad, MadjidGhittorelli, MatteoSaternus, MariolaGhosh, SohamSato, TakuichiGhoshal, SushantaSato, KimiyasuGhunem, Refat AtefSato, YoichiGiannakas, ArisSato, HidekiGiannazzo, FilippoSaumer, MonikaGianni, AretiSavaniu, CristianGiaume, DomitilleSavija, BrankoGigante, Giovanni E.Sawpan, MoyeenuddinGil, AntonioSbarufatti, ClaudioGill, PhilipScalese, SilviaGill, Richie H.S.Scalici, TommasoGillich, Gilbert-RainerScarabelli, LeonardoGiménez-Marqués, MónicaScarano, AntonioGimenez-Pinto, VianneyScarfato, PaolaGiorgio, IvanScendo, MieczyslawGirard, StevenŠčetar, MarioGiraud, StephaneSchäfer, EdgarGiri, AshutoshSchalk, NinaGirousi, StellaScharoch, PawelGiubileo, FilippoSchartel, BernhardGiuffrè, TullioScheiner, StefanGiuliani, FeliceSchennach, RobertGiunta, GaetanoScheu, ChristinaGiurgiutiu, VictorSchirp, ClaudiaGiussani, AngeloSchmidt, ThomasGizdavic-Nikolaidis, Marija R.Schmidt, Shelly JGjokas, MargaritisSchmidt, JochenGloria, AntonioSchmidt, Hans-PeterGnade, Bruce E.Schmidt, TanninGodeau, GuilhemSchmidt, RainerGoedecker, StefanSchmidt, RomyGoenuelle, YakupSchmutz, MarcGohda, EiichiSchneider, JochenGokhale, Vikrant JayantSchneider, RaphaëlGokuldoss, PrashanthSchnepf, AndreasGolis, E.Schoedel, AlexanderGoltz, Douglas M.Schöppner, VolkerGolub, IgorSchorr, MichaelGombac, ValentinaSchotter, JörgGomes, Ana P.Schubert, Th.Gomes, PedroSchuh, ChristinaGomez, David GarozSchulz, FlorianGómez De Salazar, Jose MªSchulz-Siegmund, MichaelaGómez Escobar, ValentínSchütte, BerndGomez-Flores, ReynaSchwan, MarinaGomez-Garcia, CarlosSchwartz, Benjamin J.Gómez-Soberón, José ManuelSchwarz, SimonaGomis-Bresco, JordiSchwarze, MichaelGoncalves, AntonioSchweiss, RuedigerGong, PanSchwentenwein, MartinGong, JueScoponi, MarcoGoñi, IsabelScotognella, FrancescoGonzález, SergioScott, James FloydGonzález-Carrasco, J.L.Scott, GeyerGonzález-Doncel, GasparScribante, AndreaGonzález-Fonteboa, BelénSeantier, BastienGonzález-Pérez, AlfredoSecu, MihailGonzález-Sánchez, M.I.Sedghizadeh, ParishGonzalez-Velo, YagoSedlaček, MarkoGoodall, RussellSeetharaman, SankaranarayananGooneie, AliSefat, FarshidGopalakrishnan, GopanSegawa, HiroyoGopalakrishnan, Sai GautamSegreto, TizianaGorash, YevgenSegui, C.Goraus, JerzySegura-Egea, Juan JoséGordiichuk, PavloSehaqui, HoussineGordo, ElenaSeifitokaldani, AliGórecki, TadeuszSEKINE, TomohitoGõrka, Jacek GórkaSelyanchyn, RomanGórny, MarcinSemaltianos, N.G.Gorria, PedroSemba, TakeshiGoupee, AndrewSemboshi, SatoshiGovoni, MarcoSemenova, Irina P.Graetz, KathrinSen, SanghamitraGräf, StephanSena-Cruz, JoseGrajcar, AdamSenderowski, CezaryGranet, RobertSeo, YongbeomGranollers Mesa, MartaSeo, Young-SooGranz, TonySeok, HyunGrassl, PeterSequeira, CésarGraziani, GabrielaSerban, NicolaeGraziosi, PatrizioSerra, AngelsGrazulevicius, Juozas V.Šesták, PetrGreco, AntonioSgarbossa, PaoloGreenfield, Edward M.Shacham-Diamand, YosiGreiner, ChristianShah, AashishGresil, MatthieuShah, Furqan A.Gribniak, ViktorShaha, Sugrib KumarGrigoriev, D.Shahbeigi Roodposhti, PeimanGrinblat, GustavoShahrokhabadi, ShahriarGrivel, Jean-ClaudeShalaby, MostafaGrobelak, AnnaShanjani, YaserGröger, ThomasShankar, KrishnaGrondin, FrédéricShanks, Robert A.Gruenert, WolfgangSharma, SurbhiGrydin, OlexandrSharma, YogeshGu, FanSharma, BhavyaGuareschi, RiccardoShavandi, AminGuell, Aleix G.Shaygan, MehrdadGüemes, AlfredoShemelya, CoreyGueorguiev, G.K.Shen, HaotingGuessasma, SofianeShen, Kelvin K.Gui, YilinSherburn, Jesse A.Guibal, EricShestakov, MikhailGuijarro-Berdinas, BerthaShevtsov, SergeyGuijt, Rosanne M.Shi, Shih-ChenGuilbert, DamienShi, XianmingGuillamont, DominiqueShi, Jen-BinGuimaraes, A.S.Shilko, Evgeny ViktorovichGuina, MirceaShimada, KeitaGullon, PatriciaShimizu, KazuoGuo, ZhanhuShin, YooseokGuo, XiaoaiShin, MyoungsuGupta, SanjuShinya, NorioGupta, Ram K.Shiota, TadashiGupta, AnjuShirvanimoghaddam, KamyarGupta, MohitShishkin, AndreiGuzmán De Villoria, RobertoShishkovsky, IgorGuzman-Sepulveda, Jose RafaelShofner, Meisha LeiGyawali, GobindaShokouhimehr, MohammadrezaGyörgy, EniköShortle, WalterHa, JinukShrestha, Lok KumarHa, Heon-YoungShtepliuk, IvanHa, DonhyungShu, ShipengHack, MichaelShumyantseva, Victoria V.Haddadi, FaridShunmugasamy, Vasanth C.Hadjiargyrou, MichaelShur, VladimirHAFUKA, AkiraShvartsman, VladimirHaghdadi, NimaSiad, HocineHaghighat, EhsanSiciliano, AlessioHaghshenas, MeysamSiddiq, M. AmirHahn, Konstanze R.Sidorenko, AlexanderHaider, SyedSiedlecka, Ewa MariaHaiges, RalfSiegrist, TheoHaj-Ali, RamiSilberstein, MeredithHakkarainen, MinnaSilikas, NickHalada, GarySilikas, NikolaosHald, JohnSilva, TiagoHalisch, MatthiasSilva, Francisco José Gomes DaHall, SteveSilva, HugoHallen, AndersSilva, Carlos ManuelHallett-Tapley, GenieceSilva, FilomenaHam, Hyung ChulSimka, WojciechHamasaki, HiroshiSimões, SóniaHamel, Scott E.Simonovski, IgorHampp, NorbertSimons, ThomasHampp, Norbert A.Simserides, ConstantinosHampton, Jennifer R. Pinho, LuísSimunovic, KaticaHamuyuni, JosephSing, Swee LeongHan, Chung-SoukSINGAMANENI, SRINIVASA RAOHan, Dong WookSINGH, ALOKHan, KeSingh, RanjanHanaor, DorianSingh, PrashantHanawa, TakaoSingh, HarpalHangai, YoshihikoSingh, JaiHansen, ChristopherSingh, Jitendra KumarHaque, RezwanulSingh, Pramod K.Harding, IanSingh, KalpanaHariri-Ardebili, Mohammad AminSingha Roy, SusmitHarish, SivasankaranSirutkaitis, ValdasHarmanci, Yunus EmreSivakumar, KrishnakumarHarnicarova, MartaSjoerd, RoordaHarper, JasonSkomedal, GunsteinHartl, MartinSlifka, Andrew J.Hasan, MarufSloma, MarcinHasegawa, MasashiSlonopas, AndreHasiuk, FranciszekSmallenburg, FrankHassan, Hamad UlSmalley, Joseph S.T.Hassanpour, AliSmela, ElisabethHatada, RurikoSmet, Philippe F.Hattori, TomokazuSmialek, James L.Hattori, Azusa N.Smiatek, JensHaurie, LaiaSmith, TimothyHaven, DrewSmith, MikeHawileh, Rami A.Smythe, CarlHawrylak, PawelSobkowski, MichalHayakawa, TohruSobue, TakanoriHayashi, AkariSobus, JanHayashi, YoshihitoSockalingam, SubramaniHayashi, KeiSödergård, AndersHayashi, ShotaroSoejima, TetsuroHayashi, TakahiroSofiane, GuessasmaHe, JingweiSogaard, ErikHe, JieSohn, YoungkuHeap, Michael J.Sohrabpoor, HamedHedayati, RezaSoldano, CaterinaHedhammar, MySoleimanbeigi, AliHeepe, LarsSoleimani Dorcheh, AliHegedűs, CsabaSolis-Ramos, EuripidesHegyi, AlexSolovyev, IgorHeidelmann, MarkusSoltani, NiloofarHeim, FredericSon, Jae SungHeinimann, HansSon, Seung UkHeintze, CorneliaSong, Jeong-HoonHeitkam, SaschaSong, HyunwookHekner, BartoszSong, YangHemptinne, Ferdinand DeSong, ZhiguangHenniges, UteSong, WeiminHenry, JoelSong, KenanHermoso-Orzáez, Manuel JesúsSong, XuHermouet, FabienSong, MiaoHernández, SimelysSonnier, RodolpheHerrmann, HeikoSonntag, RobertHerrmann, MathiasSontakke, Atul DHerrnsdorf, JohannesSorieul, MathiasHervás-Pérez, J.P.Sorrentino, LucaHesebeck, OlafSorrentino, AndreaHess, Dennis W.Sotor, JaroslawHidalgo, JavierSouhan, BrianHidalgo López, María Del CarmenSousa, Luis Ribeiro EHidalgo-Manrique, PalomaSovak, GuyHilloulin, BenoitSoyarslan, CelâlHintennach, AndreasSoylak, MustafaHintikka, JoukoSparavigna, Amelia CarolinaHippler, RainerSparkes, JamesHisada, KenjiSpeliotis, ThanassisHisaki, IchiroSpendier, KathrinHnida, KatarzynaSperanzini, EmanuelaHo, JohnnySpina, RobertoHo, Wen-JengSpira, Micha E.Hoang, Hong-MinhSpivak, ArthurHobbs, Michael L.Sprio, SimoneHodgson, MichaelSrinivasa, ArunHofmann, Jan PhilippSrinivasan, RaghuHofmann, AnnetteSroka, M.Hofmann, TinoStachewicz, UrszulaHofmeister, WilliamStamm, ManfredHohe, JoergStanca, Sarmiza ElenaHolby, EdwardStancanelli, Laura MariaHolmes, Natalie P.Stanzione, JosephHolynska, MalgorzataStaruch, MargoHomaeigohar, ShahinStasiuk, GraemeHong, Chang-HeeStassen, IvoHong, Soon-JikStathatos, EliasHong, JinduiStavrinadis, AlexandrosHong, JinpyoSTEFANOIU, RaduHonório, TúlioSteffen, RichardHooshmand, NasrinSteiner Petrovic, DarjaHori, YuichiroSteinmann, Wolf-DieterHorlait, DenisStepanov, N.D.Hörner, GeraldStephan, DietmarHorr, Amir M.Steriotis, TheodoreHorrillo, María CarmenSteudel, SoerenHorwat, DavidStevenson, Keith J.Hoshian, SashaStochino, FlavioHoshikawa, YasutoStöckelhuber, Klaus WernerHoskins, ClareStoić, AntunHosseini, MahsaStoyan, DietrichHosseinpour, SamanStrankowski, MichałHosseinpourpia, RezaStraumal, Boris B.Hou, JingweiStraumal, BorisHovestad, ArjanStrauss, AlfredHowell, BobStreb, CarstenHowell, CaitlinStrek, TomaszHrkac, StjepanStrelcov, EvgheniHryniewicz, TadeuszStrelniker, Yakov M.Hsiang, Hsing-IStride, John ArronHsiao, Pai-YiStrozzi, MatteoHsieh, Wen-ChuanStrube, Oliver I.Hsieh, Chien-KuoStrudwick, XantheHsieh, Wen-HsiangStrunk, JenniferHsieh, Chih-ChunStucchi, MartaHsieh, Ya-PingStyszko, KatarzynaHsieh, Pei-JuSu, Hai-ChingHsiow, Chuen YoSu, Chiung-WuHsu, Tuan-TiSu, DanHsu, Wen-dungSuárez, FernandoHsu, Fu-yinSuárez López Del Amo, FernandoHsu, Cheng-HsunSubramania, Ganapathi SHsu, ChenpengSuchanicz, JanHu, YangSudhagar, PitchaimuthuHu, HenrySudhakar, K.V.Hu, WenbingSueyoshi, KenjiHu, NanSuga, TakeoHu, ShanghsiuSugimoto, ToshikiHuang, YuandingSugino, OsamuHuang, Po-ChinSugunan, AbhilashHuang, Liang-FengSuh, Jin-YooHuang, XinyanSui, TanHuang, MengbingSukumaran, VijayHuang, YingSullivan, EirinHuang, QijinSun, LuHuang, Song-JengSun, TaoHuang, Shu-ChiSun, WenpingHuang, YiSun, Shih-JyeHuang, ShuigenSun, XiangchengHuang, Hung JiSundaram, PaulHubbe, Martin A.Sunol, Joan JosepHübers, Heinz-wilhelmSuraneni, PrannoyHueger, ErwinSURE, JAGADEESHHufendiek, AndreaSurmeneva, Maria AlexandrovnaHughes, Michael PycraftSuryanto, BennyHuh, Jung-BoSuzuki, ToshisadaHuh, Yong-MinSuzuki, KazumasaHung, Kuo-YungSuzuki, MichioHung, WayneSvane, Katrine LHuo, QinghuanSveda, MariaHurst, RogerSvensson, Ann MariHussain, BabarSwaminathan, ViswanathanHussain, N. SoorajSwiderski, WaldemarHusson, JérômeSwieszkowski, WojciechHutmacher, DietmarSwindle, AndrewHwang, Chung-JuSycheva, G.A.Hwang, Chao-LungSymes, Mark D.Hwang, Chi-SunSzabo, LorandHwang, Ki-ChulSzekely, GyorgyHwang, InchanSzeląg, MaciejHwang, DavidSzepvolgyi, JanosHwang, Seong SikSzewczyk, RomanIacomussi, PaolaTabaković, AmirIafisco, MicheleTabata, MakotoIandolo, DonataTada, NaoyaIannazzo, DanielaTadanaga, KiyoharuIanoul, A.I.Taddei, PaolaIatsunskyi, IgorTafraoui, AhmedIddir, HakimTagawa, HiroshiIgari, ToshihideTagliabue, GiuliaIglesias-Linares, AlejandroTaguchi, YoichiroIgnat, MariaTaha-Tijerina, JaimeIjiro, KuniharuTahreen, NabilaIliopoulos, KonstantinosTaiki, NakataImam, MurshidTajiri, KazuyaImperatore, StefaniaTakada, TomoyaInagaki, Y.Takagi, NoriakiInfantes Molina, AntoniaTakahashi, HidekazuInfantes-Molina, AntoniaTakahashi, YasuoInoue, GoTakahiro, KatsumiInsepov, ZekeTakaichi, AtsushiIoannidou, AlexandraTakamizawa, ToshikiIonita, PetreTakano, HajimeIordanskii, AlexeyTakashima, ToshihiroIovenitti, PioTakeguchi, TatsuyaIrie, MasaoTakemoto, ShinjiIrukuvarghula, SandeepTakeoka, ShinjiIsakov, DmitryTakizawa, ShinyaIshige, RyoheiTalaat, AhmedISHIKAWA, ToshiyukiTalebi, S. HesamodinIshizaki, KenjiTalic, BelmaIslam, KamrulTamaki, YukimichiIsland, J.O.Tamanoi, FuyuhikoIssa, AhmedTamayo, AitanaIta, KevinTamura, KenjiItkis, Mikhail E.Tan, Chung-SungIto, TaichiTan, XipengIto, KotaTANAKA, KojiIto, TakeruTanaka, KazuoItoh, TamitakeTananaiko, OksanaIvanov, Ilia N.Tanase, StefaniaIvanova, T.Tang, ZhengIwasaki, TomohiroTang, TingtingIwata, JunichiTao, SunJacak, JarosławTapie, LaurentJacot, AlainTappertzhofen, StefanJadwicienczak, WojciechTarafder, SolaimanJaecques, SiegfriedTard, CédricJafari, SajjadTarfaoui, MostaphaJahangir, IfatTarle, ZrinkaJaiswal, SwarnaTasinkevych, MykolaJajam, KailashTatay, SergioJakes, Joseph E.Tathireddy, PrashantJakiela, SlawomirTaubner, ThomasJakka, Suresh KumarTavares, AnthonyJamali, Sina S.Tavares, S.M.O.Jambovane, SachinTavares, Carlos J.Jampani Hanumantha, PrashanthTcherdyntsev, Victor V.Jamshidi, A.Tee, Benjamin C.K.Jang, SoohwanTeghil, R.Jang, ChangheuiTeixeira, P.I.C.Jang, JinahTeixeira, PauloJang, Tae-SikTEKE, AliJang, JinsungTelang, AbhishekJanicki, DamianTemiz, YukselJanik, VitTen Elshof, Johan E.Janka, OliverTena-Zaera, RamonJankauskaitė, VirginijaTeng, ChongJanovec, JozefTeo, BoonJanovszky, DoraTeramoto, YoshikuniJantunen, ErkkiTessier, NoelJavadi, AliTestoni, NicolaJaworski, MaciejTeyssier, JeremieJędrzejewska-Szczerska, MałgorzataTezvergil-Mutluay, ArzuJeeyoung, YooThakur, GarimaJefferies, Steven R.Thi Hoang Oanh, NguyenJennings, JessicaThiagarajan, GaneshJensen, EricThiele, KlausJenuš, PetraThienel, Karl-ChristianJeon, SeokwooThiéry, VincentJeon, Ju-WonThissen, PeterJeon, HyeongtagThorat, SanjayJeon, HojeongThornton, PaulJeong, Soon-JongThrivikraman, GreeshmaJeong, Doo SeokTian, FuyangJeong, JoonwooTian, YebingJesionowski, TeofilTiberti, GiuseppeJesus, AbilioTilita, George AlexandruJesus, Vazquez ValeoTimofeev, Ivan V.Jezierski, JanTimón, VicenteJha, Kshitij C.Ting, ValeskaJi, QingminTiryakioglu, MuratJi, VincentTitkov, Ilya E.Ji, TuoTkach, AlexanderJia, WeiweiTölli, HannaJia, MingtuTomac, IngridJiang, LibenTomlinson, DouglasJiang, RuinianTommasini, Steven M.Jiang, ShaohuaTomoaki, KarakiJiang, XuchuanTomoyoshi, NishiumuraJiang, LiuweiTonti, AndreaJimenez, Ana EvaTopping, TroyJiménez, NoéTorgal, F.PachecoJiménez-Aparicio, ReytesToroker, Maytal CasparyJin, XinfangToropova, AllaJin, PengTorrens-serra, J.Jin, CongruiTorres Lagares, DanielJin, FeiTorres-Carrasco, ManuelJinno, YoheiTorricelli, FabrizioJiu, JintingTosello, GuidoJjemba, Patrick K.Tosheva, LubomiraJo, WilliamToskas, GeorgiosJoaquín, Ordieres-MeréToth, Laszlo S.Jofre-Reche, Jose AntonioTotolin, VladimirJomaas, GrundeTounsi, MoncefJones, Matthew R.Tovar, NickJoos, JonasTrabia, MohamedJosé IgnacioTracy, JoeJørgensen, Jens ErikTrakakis, GeorgeJou, ShyankayTran, FabienJoung, Hyou-ArmTrifol, JonJóźwiak-Niedźwiedzka, DariaTriki, SmailJung, Hyun-DoTripathi, Jitendra KumarJuodkazis, SauliusTripodo, GiuseppeJurczak, JanuszTrueba, MonicaKabanos, ThemistoklisTrujillano, RaquelKabir, M.R.Trukhanov, Alex V.Kaczmarek, MariuszTruong, VinhKaczmarski, JakubTsai, YenlingKadhum, Mohannad J.Tsai, Huan-LiangKadib, Abdelkrim ElTsai, Che-WeiKahouli, AbdelkaderTsangouri, EleniKajitani, T.Tsao, Lung-ChuanKakiuchi, ToshifumiTsay, Chien-YieKako, TetsuyaTsay, Leu-WenKalantar-Zadeh, KouroshTseng, I-HsiangKallitsis, Joannis K.Tsipis, Athanassios C.Kalogeras, Ioannis M.Tsitsilianis, ConstantinosKalska-Szostko, BeataTsivoulas, DimitriosKamali, SaeedTsoi, JamesKamdem, PascalTsubomura, TaroKameyama, AtsushiTsubota, ToshikiKamiguchi, SatoshiTsujimoto, YoshihiroKamimura, TakafumiTsujimoto, A.Kamiński, MarcinTsukada, ShinyaKamiya, KazuhideTsukamoto, SusumuKamperidou, VasilikiTsuruoka, TakaakiKampitsis, AndreasTsutaoka, TakanoriKampouris, DimitriosTsuyumoto, IsaoKamrani, SepidehTudorache, FlorinKan, Chi-waiTuna, TaskinKanako, ShojikiTunér, JanKanda, YasuharuTung, Tran ThanhKanellopoulos, AntoniosTunuguntla, RamyaKanematsu, HideyukiTurco, AntonioKang, PilgyuTurkmen, MustafaKang, XinTurkowski, VolodymyrKang, NanTurnbull, AlanKannan, A.M.Turner, RichardKanyong, ProsperTurón, PauKao, T.S.Turski, MarkKao, Chai-LinTutunaru, BogdanKaplar, RobertTuvdendorj, DemidmaaKaraca, HalukTuzla, KemalKarami, GhodratTyagi, RajeshwarKarapanagiotis, IoannisTzounis, LazarosKarazhanov, Smagul ZhUan, Jun-YenKarg, Michael Cornelius HermannUccelletti, DanielaKargl, Rupertüçüncü, MuhammedKarjalainen, PenttiUdalov, Oleg GeorgievichKarolus, MalgorzataUddin, MohammadKarpukhina, NataliaUeda, TadaharuKarthik, ManiUemura, SeiKartopu, GirayUeno, TetsuroKarumuri, Anil K.Ueno, TasukuKasai, NaoyaUklejewski, RyszardKasap, SafaUkrainczyk, NevenKasperovich, GalinaUllah, Mohammad W.Katagiri, JunUllah, AmanKatagiri, HironoriUllah, ShatifKatarivas Levy, GalitUlrich Haselbach , PhilippKatayama-Yoshida, HiroshiUmeton, CesareKatogi, HideakiUmezu, IkurouKatsikini, M.Unnithan, Ranjith RajasekharanKatsube, NorikoUr, Soon-ChulKatsui, HirokazuUrban, PetrKatz, Jonathan I.Urbanic, R.J.Kauppinen, AnuUshak, SvetlanaKaur, SumanjeetUsui, KenjiKaushik, Nagendra K.Uzunlar, ErdalKawabata, YuichiroVaitkus, AndriusKawai, ToshiyukiValange, SabineKawakami, HiroshiValencia, ConcepciónKawamura, GoValentina, LoprestoKaynak, AkifValeo, CaterinaKazak, AndreyVallés, CristinaKazek-Kęsik, AlicjaVallianatos, FilipposKažys, Rymantas JonasValov, IliaKeane, Denis T.Van Der Auweraer, M.Kearney, CathalVan Der Meeren, AnneKeller, MichaelVan Erps, JurgenKeller, BrandisVan Herwijnen, HendrikKellesarian, SergioVan Steenberge, Paul Hm M.Kempe, HenrikVandamme, DriesKempen, KarolienVanhille, ChristianKennedy, John VVanneschi, ClaudioKennedy, FrancisVasaikar, SuhasKern, WolfgangVasconcelos, Helena CristinaKeršner, ZbyněkVasdravellis, GeorgeKeshavarz Hedayati, MehdiVasile, CorneliaKessler, OlafVautard, FredericKessler, SylviaVaz, CarlosKetov, SergeyVazquez Martinez, Juan ManuelKettwich, Sharon C.Vedarajan, Assistant Professor RamanKevadiya, BhaveshVelasco López, Francisco JavierKhaliq, JibranVelasco Ortega, EugenioKhan, M. AshrafVenäläinen, SallaKhan, FuadVendrell, XavierKhan, MuhammadVenezia, Anna MariaKhan, MohammadVenkatesan, JayachandranKhandaker, MorshedVerbeek, JosKhanna, Sanjeev K.Verdeja, Luis FelipeKhapli, SachinVerduzco, RafaelKhare, SanjayVergani, LauraKharel, ParashuVergara, DiegoKharin, ViktorVernardou, DimitraKharkar, PrathameshVervisch, BramKhatib, JamalVervoort, A.Khatibi, G.Veryazov, ValeraKheradmand, NoushaVesel, AlenkaKhirallah, KareemVicenzo, AntonelloKhodadadi, Jay M.Vidal, HilarioKhorasani, Amir MahyarVignali, ValeriaKhurshid, ZohaibVijayavenkataraman, SanjairajKichambare, PadmakarVila, AnnaKiciński, WojciechVilaseca, FabiolaKidoaki, SatoruVila-Vilela, Jose LuisKiener, DanielVillani, VincenzoKihara, NobuhiroVillanneau, RichardKillian, Manuela SonjaVillarroel, Grover ZuritaKim, Jin YoungVincent, DidierKim, BruceVinogradov, SergeyKim, Hyeong-KiVisco, AnnamariaKim, Young-KukVisintin, PhillipKim, Dae SuViswanathan, VaishakKim, Young SikViter, RomanKim, DokyoungViva, Federico A.Kim, BohyunVivo, PaolaKim, HyungsunVlahovska, Petia M.Kim, JeongyongVo, Minh D.Kim, Hwa YoungVogt, JochenKim, Bong-SungVolk, TatyanaKim, Choong-UnVolpe, GiovanniKim, Jun-HyunVon Bardeleben, H.J.Kim, Han-doVora, PatrickKim, Byung KyuVora, AnkitKim, Hee-SeokVoué, MichelKim, JanghoVrana, Nihal EnginKim, Hyun-ManVranceanu, Diana M.Kim, Seong-GonVrancken, BeyKim, Jung-GuVrsaljko, DomagojKim, Felix SunjooVuddanda, Parameswara RaoKim, HyungtakVukelic, GoranKim, Young-KwanVul, AlexanderKim, JinhoWachs, Rebecca A.Kim, Seung-JooWagner, StefanKim, Hak YongWagner, Brent T.Kim, Seon-JinWahaia, FaustinoKim, Hyun-SeokWahl, KathrynKim, See JoWaite, Matthew M.Kim, Jung HoWakabayashi, YusukeKim, Do YoungWaki, KeikoKim, HoyeolWakisaka, MinatoKim, Hyun JaeWaldmann, DanieleKim, Dong-Joo (Daniel)Walock, MichaelKim, Jae-HyunWalsh, William R.Kim, KyungmokWalsh, Michael T.Kim, Jin-YeonWalsh, FrankKim, YangWalsh, LaurenceKim, JihanWalter, RhysKim, JongbeomWaltham, ChrisKim, Byeong HeeWalther, FrankKim, SeyoungWan, ChaoyingKim, Si JoonWang, ShaopengKim, YoongWang, Chiu-YenKim, Hak-YongWang, XiaomingKIMURA, Shin-ichiWang, LiangKimura, KunioWang, ZhiguangKing, Sean W.Wang, ZheKing, DerekWang, YueKirane, KedarWang, Yi-ChengKirillov, AlexanderWang, YunxiaoKisała, PiotrWang, FenglinKisiel, RyszardWang, Yun-CheKiss, JánosWang, XiaoyongKissin, Yury V.Wang, YanKitagawa, HiroyukiWang, XufengKitahara, TatsumiWang, JianyunKitamura, ChiakiWang, DongKitamura, MichihideWang, Peng-Yuan (George)Kityk, AndriyWang, LiliangKivisaari, PyryWang, PangpangKlammer, AngelikaWang, FukeKlaudia, LichtenbergWang, MingchaoKlemczak, BarbaraWang, HuaqingKlemperer, Walter G.Wang, XiaohongKlimek, KatarzynaWang, QishengKliros, George S.Wang, YunKlöcker, HelmutWang, QiguiKlöden, BurghardtWang, DanlingKlose, ChristianWan-Wendner, RomanKlotzkin, DavidWard, LiamKnapek, MichalWark, MichaelKnite, MārisWarren, Robert J.Knupp, GerdWatras, AdamKo, Bao-TsanWeber, LudgerKo, Yong-HoWeber, Franz E.Ko, Young GunWegehaupt, FlorianKobata, JunpeiWegrzyn, MarcinKobayashi, MakikoWei, ZhishunKobayashi, MotoyoshiWei, HuiliangKoblischka, Michael RudolfWei, YangKoci, KamilaWeigand, R.Koduru, Janardhan ReddyWeigand, AnnikaKoenigsberger, ErichWeinberger, ChristianKohlmann, HolgerWeise, JörgKoida, TakashiWeiß, SabineKoinkar, Dr. PankajWeissenrieder, JKõiva, RistoWendrock, HorstKoizumi, HiroyasuWeng, XuanKok, R.J.Weng, YiKolan, KrishnaWerner, Jürgen H.Kolbe, JanaWharton, JulianKolenda, ZygmuntWheeler, Dean RKolerman, RoniWhiting, MarkKollenberg, WolfgangWibowo, DavidKolmas, JoannaWięckiewicz, MieszkoKomarasamy, MageshwariWiens, MatthiasKomsa, Hannu-PekkaWierichs, RichardKonarova, MuxinaWiesbrock, FrankKonda Gokuldoss, PrashanthWijayasundara, MayuriKondo, ToshiakiWilde, JürgenKondo, TakahiroWilén, Carl-EricKonegger, ThomasWilhelm, StefanKong, QingzhaoWillander, MagnusKong, XiaohuaWillberg, ChristianKonosu, ShinjiWillem, Visser ClaasKonstantinov, KonstantinWilliams, Kathryn R.Kontic, RomanWillis, David A.Kontou, EviWilson, LeeKoo, Yang-HyunWilson, Benjamin P.Kootala, SujitWinter, AndreasKoplin, ChristofWirth, Christopher LKorampally, VenumadhavWisniewski, AdamKordesch, Martin E.Wistuba, MichaelKoricho, Ermias GebrekidanWitkowska Nery, EmiliaKortschot, Mark T.Witkowski, ArturKostryzhev, Andrii G.Wittmann, FolkerKoter, StanislawWittmar, AlexandraKotoka, RubenWojciechowski, ŁukaszKotousov, AndreiWojewoda-Budka, JoannaKotsakis, Georgios A.Wolcott, Paul J.Kotsiomiti, EleniWolfe, Tonya B.Koumoulis, DimitriosWoll, ChristofKoumoulos, EliasWondraczek, KatrinKouris, LASWong, Joanna C.H.Koutsogeorgis, DemosthenesWong, Lai MunKoutzarova, TatyanaWong, Man HoiKováč, JaroslavWong, HongKovacic, HervéWood, DelilahKovalchenko, Andrii M.Woodward, DavidKovalcik, AdrianaWoszuk, AgnieszkaKowalczuk, Marek M.Wróblewski, GrzegorzKowalczyk, Krzysztof KarolWu, Shih-ChingKowalik, PawełWu, Sheng-ChiKowalska, EwaWu, WeidaKowalski, KarolWu, XijiaKowalski, KonradWu, Chin-SanKoziel, JacekWu, Sheng YunKozinov, SergiiWu, QinglinKozyra, PawełWu, ChengtieKrajewski, WitoldWu, Tsung-TaKramer, PhillipWu, Kevin C.-W.Krantz, ClaudeWu, QiKranz, StefanWuu, Dong-SingKranzmann, A.Xavier, JoséKrasnolutsky, SergeyXenofon, StrakosasKrastev, RumenXi, WeixianKratzer, PeterXiang, ChunhuiKraus, TobiasXiao, ZhigangKrenner, HubertXie, KunpengKrim, JacquelineXie, JiulongKrishna, LakshmiXotta, GiovannaKrishnaswamy, KirubaYaacoubi, SlahKriston, AkosYagami, KimitoshiKřivý, VítYahiaoui, Riad.Krolczyk, GrzegorzYahiaoui, RiadKrólicka, AgnieszkaYamabe, JunichiroKroll, PeterYamaguchi, HiroyukiKrumeich, FrankYamaguchi, SeijiKrzanowski, James E.Yamaguchi, SatoshiKsiazek, MarzannaYamamoto, NamikoKtena, AphroditeYamane, HidekiKu, ZahyunYan, XingzhongKu, Kang MoYan, XueliangKubiak, TomaszYan, QiminKubo, MasatakaYan, NingKudela, PawełYanagida, SayakaKuhn, Matthew R.Yanagishima, TaikiKujawa, Joanna AgnieszkaYanase, KeijiKulagin, RomanYandouzi, MohammedKulkarni, NehaYang, Su-HuaKum, Kee-YeonYang, Te-HsinKumada, NobuhiroYang, Shou-YinKumar, RajYang, Ta-I.Kumar, ManishYang, RuiguoKumar, SubhalakshmiYang, YongKumar, AlokYang, Chung-WeiKumar, AdityaYang, MijiaKumaresavanji, MalaivelusamyYang, Ru-YuanKun, RobertYang, JamesKundin, JuliaYang, FeiKundu, JoydipYang, Chong KuangKundu, ManabYang, MinyangKunimine, TakahiroYang, ChaoKuntz, MatthiasYang, Kai-ChiangKunze, KarstenYang, XiaodongKuo, Wen-TenYang, Jen-ChangKUO, MENG-IYang, Hyun KyoungKupka, MarianYao, LiKurek, AndrzejYao, TiankaiKurlyandskaya, Galina V.Yao, HiroshiKuroshima, ShinichiroYao, JianKuttner, ChristianYapici, Murat KayaKuuskeri, JaanaYastrebov, Vladislav A.Kuwano, NoriyukiYasuhara, RyoKwak, Moon K.Yasui, ShintaroKwon, Hyuck-InYasui, KanjiKwon, Tae-yubYavin, EphraimKwon, Nam HeeYe, HuaiyuKyaw, Si ThuYeager, JohnKynicky, JindrichYeaman, JohnKyritsis, PanayotisYeh, Chih-koKyzas, George Z.Yeh, Chun-LiangKyziol, KarolYeh, Jui-MingLa Spada, LuigiYeh, Yin-TingLabisz, KrzysztofYeih, Wei-ChungLadavière, CatherineYEN, HUNG-WEI (HOMER)Ladero, MiguelYeo, In-SungLagos, Miguel AngelYeo, JunyeobLagugne-Labarthet, FrancoisYeo, Jong-SoukLahmer, TomYeong, WaiLai, Chien-ChihYerra, NarendranathLainé, Philippe P.Yi, LongLakshminarayanan, RajamaniYi, JiabaoLalevée, JacquesYi, JaeseokLalwani, GauravYildiz, BilgeLambert, PaulYilmaz, TurgutLambiase, FrancescoYin, YanchunLambrecht, WalterYokozeki, TomohiroLampropoulos, AndreasYoo, ByungseokLancaster, RobertYoo, ChungsikLanceros-Méndez, SenentxuYoon, SonghunLane-Serff, GregoryYoon, Kee BongLangone, RoccoYoon, Ji-WookLanzi, MassimilianoYoon, MinjoongLao, Ka UnYoshihara, HiroshiLappan, UweYoshitake, IsamuLara-Curzio, EdgarYou, Nam-HoLargitte, LucieYou, Chun-YeolLarouche, DanielYou, Tae-sooLasheras, AndoniYoung, DavidLass, EricYoungblood, Jeffrey P.Lattuada, MarcoYounis, AdnanLau, DenvidYousefi, Azizeh-MitraLaukkanen, PekkaYoza, Brandon A.Laurenti, MarcoYu, Rena C.Laurson, LasseYu, ZiniuLavella, MarioYu, LeonLaw, D.W.Yu, XunLayani, MichaelYu, Hak KiLázár, IstvánYu, TaekyungLazaro Nebreda, JaimeYu, Jae WoongLazzara, GiuseppeYu, ZhengshanLe, Quoc ToanYu, AiminLe Bihan, FranceYu, QingliangLe Moigne, NicolasYuan, JihuiLe Saux, VincentYue, WeishengLebedkin, Sergei F.Yun, HongseokLeblebicǐoǧlu, BinnazYusenko, Kirill V.Lee, Li-TingYvonnet, JulienLee, Jae ShinZago, MatteoLee, Kee SungZajickova, ZuzanaLee, SejoonZamanzade, MohammadLee, Hyuck MoZammit, AnnLee, H.-M.Zaniewski, John P.Lee, Jae MyungZanotti, CristinaLee, MinheeZanotto, F.Lee, WoongZawadzki, JaroslawLee, Seok-WooZaytsev, DmitryLee, Chih-HaoZehnder, MatthiasLee, Byung ChulZekonyte, JurgitaLee, Kang TaekZeng, JuqinLee, Bang YeonZervaki, Anna D.Lee, Seok-JaeZhai, YuweiLee, Chang YeonZhan, HaifeiLee, Chang-HoonZhan, NaiqianLee, Jin-KyunZhang, HongtaoLee, SunwooZhang, LaichangLee, Koon-YangZhang, Wenjun (Chris)Lee, SunghwanZhang, Xueqiang AlexLee, Kyoung G.Zhang, WeiLee, Cheng-chungZhang, XieLee, ByeongchanZhang, RunLee, Chia-hungZhang, YanLee, Sang-JoonZhang, KuangyuanLee, Mon-JuanZhang, LiangLee, JooyoungZhao, YajunLee, Sang-BaeZhao, ZhenghangLee, MinhyungZhao, Meng-QiangLee, Jea UkZHAO, XINXINLee, Young-ChulZhao, JingweiLee, Dai SooZhao, JingzhouLéger, BastienZhao, ShanLegutko, StanislawZhao, XiaoaiLeinenbach, ChristianZhao, BoxinLeipner, Hartmut S.Zheng, LiqiuLemu, Hirpa G.Zheng, PaigeLengliné, OlivierZhitova, Elena S.Leonora, VellemanZhmud, BorisLepadatu, SerbanZhou, HuanLeparoux, MarcZhou, WenzhongLeporatti, StefanoZhou, J.Lesch, AndreasZhou, BichengLesiak, PiotrZhou, ShengqiangLessard, BenoitZhou, YuanyuanLetcher, ToddZhu, PeifenLevy, StevenZhu, WeiLi, GangZhu, YakunLi, MeichunZhu, ChunhongLi, YapingZhukovskyi, MaksymLi, JichaoZielińska-Jurek, AnnaLi, ShengxiZielinski, AdamLi, LongyuZietz, CarmenLi, LuluZiewiec, KrzysztofLi, WeiZin, ValentinaLi, PeterZinna, FrancescoLi, XiaodongŻmojda, JacekLi, JiehuaŻmudzki, JarosławLi, QinZocca, AndreaLi, Ji-guangZoidis, PanagiotisLi, ZhongjingZoppellaro, GiorgioLi, MingdaZornoza, EmilioLi, KaichangZou, Fangxin (Frank)Li, JiashenZozulya, AlexeyLi, QiliangZubkova, ValentinaLi, ZibiaoZucchi, FabrizioLi, DanZuo, FeiLi, YujiaoZürch, MichaelLiang, Tsair-ChunZwaan, FrankLiang, FengZyła, GawełLiang, Xihui


